# Supplementary material for: A Facile and Efficient Protocol for Preparing Residual-Free Single-Walled Carbon Nanotube Films for Stable Sensing Applications
Source: Nanomaterials (Basel). 2019 Mar 21;9(3):471. doi: 10.3390/nano9030471 (PMC6474193; doi:10.3390/nano9030471)
Supplement: Supplementary file 1 [file nanomaterials-09-00471-s001.pdf]

nanomaterials

Appendix A

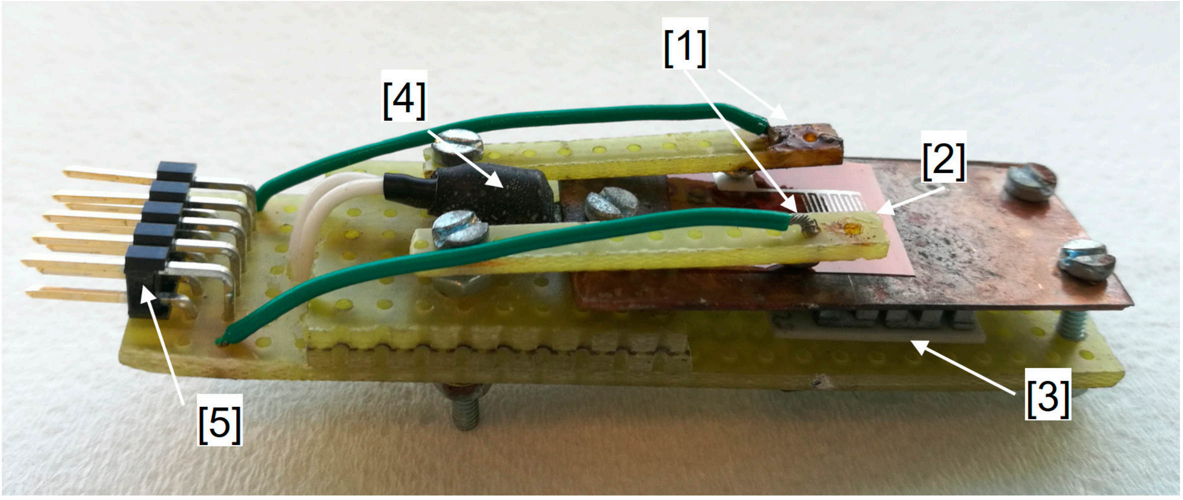

Figure S1. Photograph of the sensor module with a CNT-based sensor mounted to it. For the IDE structure, screen printed silver electrodes were used, whereas for the substrate, polyimide was used. The sensor module consists of [1] contact leads to form an electrical connection to [2] the sample, [3] a Peltier element to control the temperature, [4] a Pt100 thermoresistor for in-situ temperature monitoring and [5] contact pins for the data acquisition.

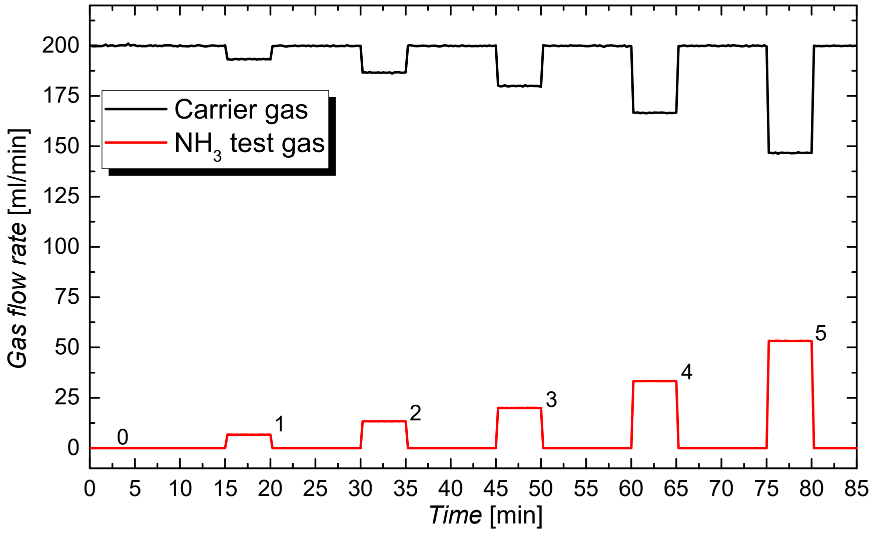

Figure S2. Gas flow rates for the nitrogen carrier gas and the ammonia test gas over time. The moments at which the concentrations of the gases are changed are labelled consecutively from 0-5 for NH_3 concentrations of 0, 10, 20, 30, 50 and 80 ppm, respectively.

Summary for the relative sensitivity factors (RSF) for the relevant elements presented in this work.

Table S1. Summary for the relative sensitivity factors (RSF) for the relevant elements presented in this work.

Element & line	RSF
C 1s	0.25
O 1s	0.66

Na 1s	2.3
N 1s	0.42
Si 2s	0.26

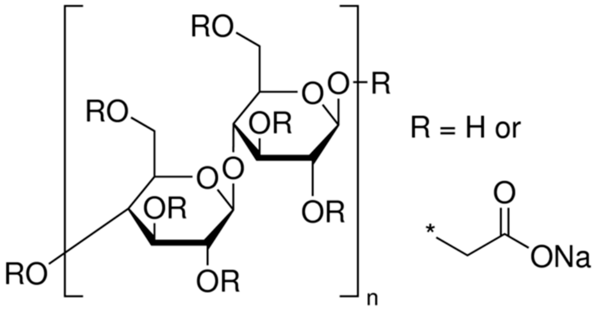

Figure S3. Structural formula for the sodium carboxymethyl cellulose monomer with the chemical formula $C_{28}H_{30}Na_8O_{27}$.

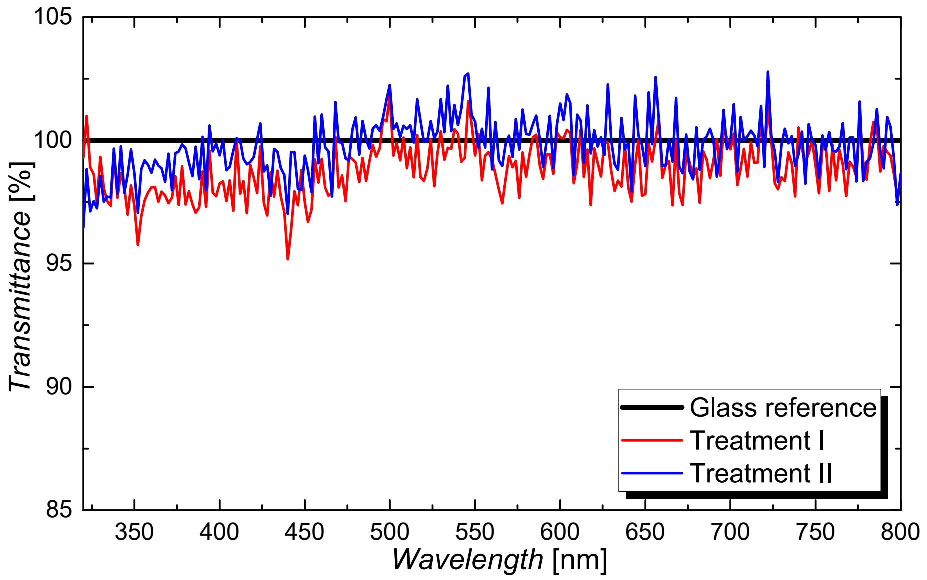

Figure S4. Transmittance spectra of the CMC-CNT films over a wavelength region of 320 to 800 nm for the films subjected to treatment I and treatment II, in accordance with the description provided in Sec. 3.1. The bold solid line represents the reference transmittance spectrum for the glass spectrum that is defined as 100% transmission for all wavelengths.
